# A cross-species protocol for ultrasound-guided intrauterine injections across gestation

**DOI:** 10.64898/2026.07.07.737050

**Published:** 2026-07-11

**Authors:** Ana Rita Ribeiro Gomes, Natalie Hamel, Surjeet Mastwal, David C. Ide, Kuan Hong Wang, David A. Leopold

**Affiliations:** 1 Section on Cognitive Neurophysiology and Imaging, Systems Neurodevelopment Laboratory, National Institute of Mental Health, National Institutes of Health, Bethesda, MD, 20892, USA; 2 Section on Instrumentation, National Institute of Mental Health, National Institutes of Health, Bethesda, MD, 20892, USA; 3 Department of Neuroscience, Del Monte Institute for Neuroscience, University of Rochester Medical Center, Rochester, NY, 14642 USA; 4 National Institute for Neurological Disorders and Stroke, National Institutes of Health, Bethesda, MD, 20892, USA; 5 These authors contributed equally; 6 Senior authors; 7 Technical contact; 8 Lead contact

## Abstract

This step-by-step protocol provides a cross-species, non-surgical approach that enables prenatal gene delivery to the developing nervous system in rats and marmosets. Under transabdominal ultrasound guidance, intracerebroventricular injection of recombinant adeno-associated virus vectors into the fetal brain achieves robust and long-term transduction from prenatal stages into adulthood. This approach can be adapted to other species and target sites outside nervous system, enabling safe and selective intrauterine manipulation and the generation of diverse experimental models for basic and preclinical research.

For complete details on the use and execution of this protocol, please refer to Ribeiro Gomes et al (2026)^1^.

## Graphical abstract

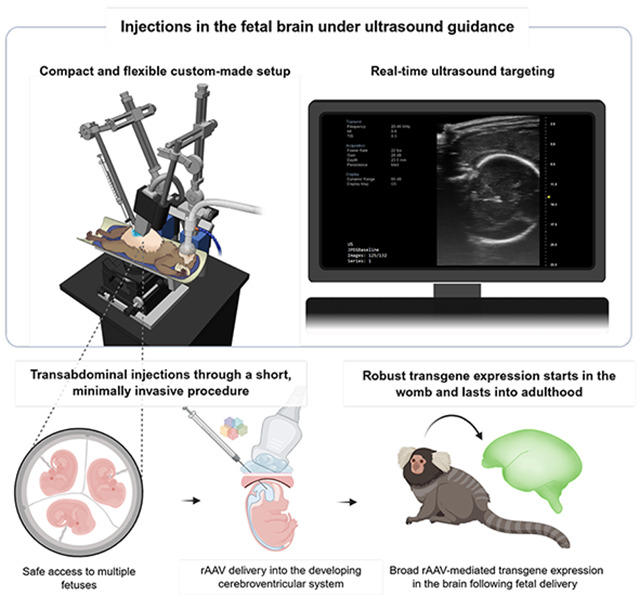

## Before you begin

Experimental procedures during gestation allow researchers to study developmental processes, including how manipulations of the fetus and its intrauterine environment influence biological outcomes. Ultrasound imaging guidance greatly facilitates such interventions by providing safe and targeted access to fetal compartments, including for prenatal gene delivery to developing neural cell populations. Critically, delivery of recombinant adeno-associated viruses (rAAVs) into the cerebrospinal fluid (CSF) of developing animals enables widespread gene transfer across the brain. The efficiency and distribution of transduction are strongly influenced by developmental stage, making the timing of delivery an important experimental variable. In altricial species such as mice, major developmental processes, including cortical lamination and the establishment of long-range connections, begin prenatally but continue throughout early postnatal life. In primates, however, development is more advanced at birth, and many equivalent developmental events are shifted to the prenatal period. Consequently, developmental stages that can be targeted postnatally in mice require prenatal access in primates. Here, we present a step-by-step protocol for ultrasound-guided fetal intracerebroventricular viral injection (FIVI) of rAAV in marmosets (Callithrix jacchus) and rats (Rattus norvegicus). The procedure was initially developed and optimized in rats before being translated to marmosets, small New World primates that share key developmental, anatomical, and functional characteristics with humans. Together, these models illustrate the cross-species applicability of the approach, while providing gene delivery strategies for both a genetically tractable rodent model and a translationally relevant nonhuman primate. FIVI enables broad gene transfer and stable, long-term transgene expression in wild type animals, facilitating the generation of complementary quasi-transgenic models for research and translational applications from prenatal development through adulthood.

### Innovation

The key innovation of this protocol is the ability to perform fetal injections in a nonhuman primate model through a short, minimally invasive, non-surgical procedure, addressing the longstanding challenge of safe and targeted access during gestation. A custom mechanical setup stabilizes the injection trajectory, while electrocautery-assisted entry facilitates penetration of maternal tissues with minimal displacement of the fetus. Combined with ultrasound guidance, this approach enables selective targeting of fetal structures while avoiding laparotomy, uterotomy, and direct manipulation of the uterus or fetuses. Although such invasive procedures are commonly performed in rodents, they carry significant risks for both the primate dam and fetus, including miscarriage. While procedural outcomes can be improved ^2-4^, risk mitigation typically requires specialized training and large numbers of animals. In primate models, these constraints have historically limited the adoption of fetal manipulation approaches and, consequently, restricted investigations of developmental processes that are highly relevant to human biology and disease.

As an additional feature, although this protocol focuses on CSF injections, the approach can be adapted to target tissues beyond the fetal nervous system, including other fetal organs and components of the intrauterine environment such as the amniotic cavity and placenta. Initially developed in rats and subsequently adapted to marmosets, it establishes a cross-species framework for ultrasound-guided intrauterine manipulation and gene delivery that can be extended to additional species, thereby expanding the pool of genetically engineered models available for research. Here, we describe the overall procedure and specify steps required for fetal CSF injections in the two models used, marmosets and rats.

### Institutional permissions

All procedures were approved by the Animal Care and Use Committee (ACUC) of the Intramural Research Program of the National Institute of Mental Health (NIMH) and performed at the U.S. National Institutes of Health in accordance with institutional guidelines.

### Preparation of the pregnant dam

#### Timing: Prior to procedure

1. Female marmosets are continuously paired with males for breeding in stable family groups and are not hormonally synchronized. Timed-mated rats are either purchased from an approved vendor or bred in-house.

***Note***: In Ribeiro Gomes et al. (2026), time-mated rat dams were purchased from Charles River Inc. They were singly housed upon arrival with gestational ages varying between postconception (PC) day 3 and PC12, and acclimated to the vivarium for at least 72h before the procedure.

***Note***: For in-house breeding, adult female rats (≥8 weeks old) are synchronized by intraperitoneal injection of 0.2 mL of a 0.4 mg/mL solution of [des-Gly10, D-Ala6]-LH-RH ethylamide acetate salt hydrate between 10:30 and 11:00 AM (12 h light/12 h dark cycle). A stock solution (5 mg of hormone in 1.25 mL 1× PBS) is aliquoted and stored at −20°C. A fresh working solution is prepared on the day of injection by thawing the stock at 4°C and diluting to 0.4 mg/mL with sterile normal saline; unused working solution is discarded. Females are paired with males 96 h after the hormone injection, ideally between 11:30 AM and 12:00 PM. Vaginal plugs are checked the following morning before 9:00 AM. Breeding efficiency improves when males are single-housed and rested (not actively mating) for at least 1 week prior to pairing.

***Note***: Since marmoset dams are not timed by hormone cycling, pregnancy is detected by transabdominal ultrasonography and monitored at least once monthly using the FUJIFILM VisualSonics Vevo MD UHF22 and UHF48 linear transducers. The females are hand restrained to minimize movement and given a positive food reward before, during, and after each scan. The ventrodorsal and transverse diameter of the uterus and uterine lumen, along with the biparietal diameter of fetus skull are measured. The postconception (PC) dates for the procedures are estimated using the published values from Oerke et al. (1995)^5^. Subsequently, these dates are re-estimated after birth by subtracting 143 days from birth, accounting for the average pregnancy length in marmosets, between 140 and 145 days ^5^.

### Preparation of the viral vector

#### Timing: Prior to procedure

2. Upon receipt, the viral vector encoding the gene of interest is stored at −80°C until use.

### Preparation of sterile FIVI instruments

#### Timing: Prior to procedure

3. Sterilize all procedural instruments – needles, needle guides, syringes, filtered pipette tips, curved hemostatic forceps, ball end hex driver 5/64• and # 3/32 – using an autoclave or ethylene oxide gas sterilization.
4. Verify that all procedural drugs (see Key Resources Table) and consumables – sterile and non-sterile tongue depressors, sterile and non-sterile cotton swabs, gauze, sterile and non-sterile ultrasound gel, disinfectant wipes, return electrode – are available and within their expiration dates.
5. Prepare the setup equipment and disinfect external surfaces by wiping with sodium hypochlorite-based disinfectant wipes.

***Note***: The disinfectant used should be selected in accordance with the institutional guidelines and facility-specific standard operating procedures.

## Materials and equipment

**Figure 1.**
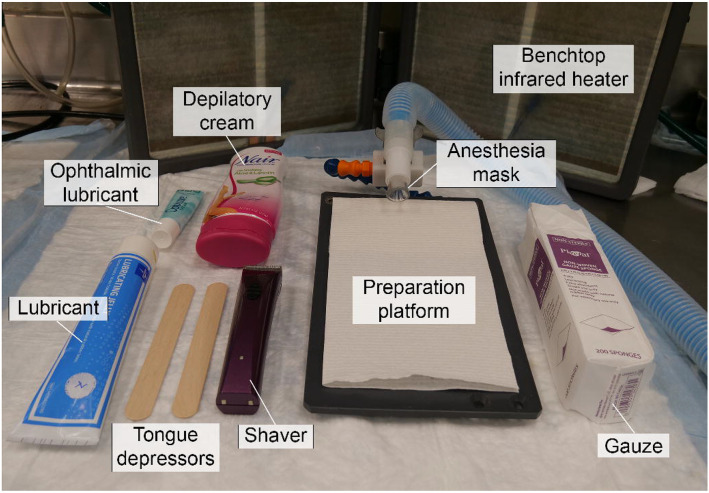
Materials and setup for preparation of the abdominal region of the dam. The preparation area includes a platform with support for stabilizing the anesthesia tubing over the dam’s mouth and/or nose while in the supine position. Tools and consumables for ophthalmic lubrication, trimming, and, when necessary, depilating the abdominal fur or hair are kept readily available. Following abdominal preparation, the animal is repositioned into the prone position for removal of a small dorsal fur patch to prepare for return electrode placement at the FIVI platform.

**Figure 2.**
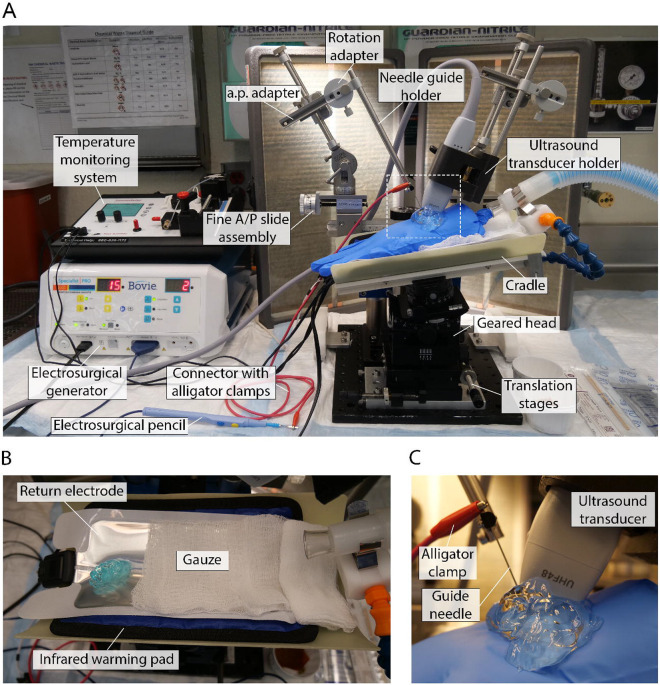
Materials and experimental setup for ultrasound-guided fetal intracerebroventricular viral injection (FIVI). The FIVI experimental area includes the injection platform, heating elements, physiological monitoring system, and electrosurgical unit (panel A). Following transfer from the preparation platform, the pregnant dam is positioned supine on the FIVI cradle over the return electrode (panel B). The electrode is covered with gauze except at the contact site with the shaved dorsal patch, and conductive gel is applied to ensure proper current return and minimize tissue injury. After stabilization in the cradle, the sterile field is prepared, and sterile ultrasound gel is applied over the abdomen. Only sterile or disinfected items contact the sterile field, including cotton-tipped applicators, tongue depressors, the guide tube and injection needle, and the ultrasound transducer. Using the translation stages and geared head, the target fetus is positioned beneath the ultrasound transducer. The fine anterior/posterior (A/P) slide assembly and A/P adapters allow precise positioning of the guide tube and transducer during fetal targeting. Once aligned with the transducer, the guide tube is advanced through the ultrasound gel toward the skin (panel C). Electrocautery-assisted entry is achieved through an alligator clamp connected to the exposed non-insulated portion of the guide tube (panel C), enabling skin penetration with minimal to no fetal displacement (see also Methods Video 2).

Key materials and equipment required for the protocol are shown in Figures 1 and 2 and listed in the Key resources table. Drawings of the guide tube holder and cradle are available in **Data S1**.

**Figure 3:**
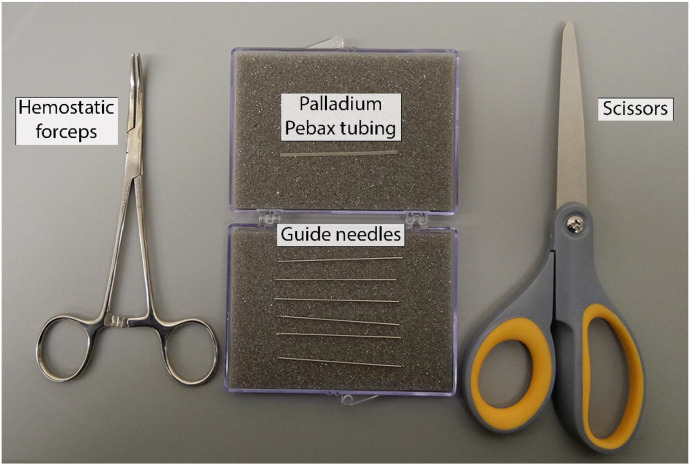
Materials used for sheathing guide tube needles.

Once received, the custom-made guide needles (hubless Hamilton Company needles) are cleaned with acetone followed by 70% ethanol. Once dried, they are sheathed with Palladium Pebax Heat Shrink Tubing. Figure 3 shows the materials used.

**Figure 4:**
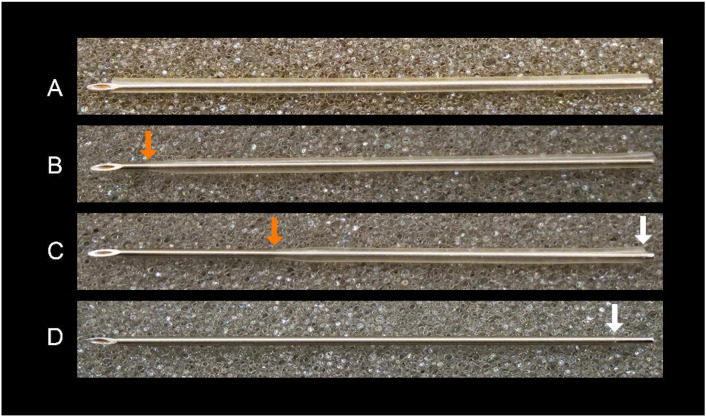
Palladium Pebax tubing contraction during guide needle sheathing. Panel A shows positioning of the tubing over the guide needle, leaving approximately 3 mm (~0.12 in) of the needle exposed at the beveled end. Panel B shows initial contraction of the tubing beneath the bevel following heat application with a heat gun. Panel C shows further contraction of the tubing along the beveled end, resulting in retraction of the tubing from the blunt end toward the beveled end. Panel D shows the guide needle after complete tubing contraction. In panels B and C, orange arrows indicate the transition between contracted and non-contracted tubing. In panels C and D, white arrows indicate the tubing edge at the blunt end, showing progressive tubing retraction and the remaining exposed segment used for hemostatic forceps handling in C and alligator clamp attachment in D.

The guide needles were sheathed using the following steps (see **Methods Video 1**):

1. Cut the Pebax tubing to a length of approximately 46 mm (~1.8 in).
2. Position the tubing over the guide tube, leaving approximately 3 mm (~0.12 in) of the needle exposed at the blunt end (Figure 4A). Maintain a distance of 1-2 cm between the heat source and the tubing during heating.
3. Using a heat gun (temperature setting: 300°C or 570°F), manually holding the guide needle and tubing, apply heat to the beveled end of the needle until the tubing is securely attached (Figure 4B). Ensure that the tubing is positioned immediately below the bevel to minimize current loss during cauterization.
4. Once enough non sheathed needle length is available at the blunt end of the needle (Figure 4C), hold the needle with hemostatic forceps. Gradually move the heat source from the beveled end toward the blunt end over approximately 10-20 seconds while continuing to apply heat until the tubing is fully contracted around the needle (**Methods Video 1**; Figure 4D). This allows the tubing to contract uniformly along the length of the needle and prevents the formation of uncoated segments that may impair current conduction through the needle.

**Alternatives**: The Clarius L20 HD3 portable handheld ultrasound system can be used as an alternative to the Vevo MD ultrasound system (compare Methods Videos 2 and 3). Although image resolution is lower than that of the UHF48 transducer, which we use for most injections, and slightly lower than that of the UHF22 transducer, the device provides sufficient image quality for pregnancy confirmation, gestational staging, and FIVI. Its substantially lower cost may facilitate adoption of this protocol by a broader range of research groups.

**Alternatives**: To further reduce costs, the vertical translation stage (VAP4/M, KOPF) carrying the A/P bar (KOPF) can be omitted. Although this component provides additional flexibility for adjusting the distance between the dam and the guide or ultrasound transducer holders, these adjustments are infrequently required. Instead, the A/P bar can be mounted directly onto longer metal rods attached to the breadboard. If additional vertical distance is required, a longer metal rod can be used to carry the guide holder and ultrasound transducer.

## Step-by-step method details

### Preparation of pregnant dams for FIVI procedure

This section describes all pre-procedural preparation steps necessary for efficiently and safely perform the FIVI procedure, including: (1) transport to the procedure room and fasting (marmosets only); (2) anesthesia induction and maintenance; (3) physiological monitoring setup; (4) administration of analgesia and perioperative medications; (5) hair removal to improve echogenicity; (6) return electrode preparation; (7) positioning of the dam; and (8) sterile field creation.

#### Timing: 20-30 min

1. Set up the room with the tools, consumables, setup and ultrasound machine (Figures 1 and 2).
2. Turn on the support heating elements before transferring the pregnant dam to the procedure room.

***Note***: For both marmosets and rats, we used multiple heat sources while preparing the pregnant dam, including an infrared warming pad, a benchtop infrared heater and an overhead heating lamp. Continuous heating was provided by the warming pad, with supplemental heat from the other sources applied as needed to maintain body temperature between 37-38 °C.

3. Transfer the pregnant dam from the housing room to the procedure room.

**CRITICAL**: For marmosets, perform the procedure after a fasting period of at least 4 hours to minimize the risk of regurgitation and aspiration during anesthesia. This timing might change in accordance with the institutional guidelines and facility-specific standard operating procedures. In our case, most procedures were performed following overnight fasting (approximately between 12-14 hours) without complications.

4. Weigh the pregnant dam and administer premedication as appropriate.
  - For marmosets, administer diazepam (0.25–1.0 mg/kg, 5 mg/mL; i.m) as a sedative and muscle relaxant, and allow 10-15 min for absorption prior to induction of anesthesia. Monitor for excessive sedation and respiratory or cardiovascular depression.

***Note:*** Premedication is administered only to marmosets.

***Alternatives:*** Alternative sedative or pre-anesthetic regimens approved by the relevant Institutional Animal Care and Use Committee (IACUC) may be used.

5. Induce anesthesia with 3% isoflurane delivered at a flow rate of 1 L/min.

***Note:*** For marmosets, induce anesthesia using a face mask (VetEquip Mask #3 or Mask #4). For rats, perform induction in an anesthesia induction chamber.

6. Once the dam is anesthetized, transfer the animal to the preparation setup (Figure 1) and place it in the supine position. Monitor respiratory rate and assess the toe pinch reflex to confirm adequate depth of anesthesia.

***Alternatives:*** A reusable heat pack (e.g., Arbor Scientific, see Key resources table) can be used in this step. However, careful temperature monitoring is required to prevent overheating and thermal injury to the dam.

7. Attach the anesthesia mask tubing to the anesthesia tubing holder in the preparation setup and deliver the anesthetic agent using species-specific mask configurations. In marmosets, the mask (Drager anesthesia mask) is stabilized over the marmoset face, covering both mouth and nose. In rats, isoflurane is delivered through a fitted nose cone stabilized over the rat nose.

***Alternatives***: The procedure cradle may be used for both animal preparation and ultrasound-guided injection to maintain stable anesthesia and consistent temperature regulation.

8. Reduce the isoflurane concentration and maintain anesthesia at 1-2% for marmosets and 1-3% for rats.
9. Set up the physiological monitoring system and initiate continuous monitoring of vital (cardiovascular, respiratory, and central nervous system) functions.

***Note:*** Use a physiological monitoring system to monitor pulse oximetry and regulate body temperature (marmoset: IntelliVue MX500; rat: Kent Scientific SomnoSuite or PhysioSuite). In marmosets, additionally monitor end-tidal CO^2^, respiratory rate, and heart rate. Provide supplemental heating using a benchtop infrared heater and an overhead heating lamp to maintain body temperature between 37-38°C and prevent hypothermia.

**CRITICAL**: Monitor vital functions, and toe pinch reflex throughout the procedure and record values every 10 min. Manually assess respiratory rate to ensure appropriate isoflurane delivery and prevent anesthetic overdose. Assess proper mucous membrane color. At minimum, monitor temperature and SpO^2^ in rats and marmosets. In marmosets, we recommend additional monitoring of end-tidal CO^2^. Adjust the isoflurane concentration as needed (e.g., in response to slow or rapid breathing), and pause the procedure until physiological parameters stabilize if necessary.

10. Administer perioperative medications.
  - In marmosets, administer meloxicam (0.1–0.2 mg/kg, 5 mg/mL; i.m or s.q.) for preemptive analgesia, and atropine (0.2 mg/kg, i.m.) to reduce salivary/respiratory secretions.
  - In rats, administer meloxicam (1 mg/kg; 1.5 mg/mL; i.m or s.q.) for preemptive analgesia.

***Alternatives:*** Other agents approved by the relevant IACUC may be used.

11. Apply ophthalmic lubricant to both eyes to prevent corneal drying.
12. Trim the abdominal fur and apply depilatory cream to eliminate the undercoat. Apply the depilatory cream to the abdominal surface, immediately caudal to the last ribs. For later gestational stages, extend the application to the lateral aspects of the abdomen to facilitate access to fetuses positioned laterally.

**CRITICAL:** When trimming the abdomen of the rat dam, pay special attention to the nipples to prevent injury. If needed, mark the placement of the needles with a pen for better retrieval. In both rat and marmosets, carefully control the application time of the depilatory cream to prevent chemical burns. Apply a thick layer of cream to the shaved area. After 30 seconds, begin removing the cream using a tongue depressor to assess hair removal. If necessary, reapply the cream in 10 segments until hair is fully removed. Remove the bulk of the cream with a tongue depressor, then gently clean the area with warm water or saline using a compress to eliminate all residues and prevent skin lesions. Do not use alcohol to remove the cream, as this may cause skin irritation.

***Note:*** In marmosets, depilatory cream is not always required. When needed, it is typically used only for the first procedure. In subsequent procedures, adequate echogenicity is usually achieved by trimming the fur alone.

13. Reposition the animal in the prone position and trim a small fur patch (approximately 2 × 3 cm) on the dorsal surface, anterior to the tail base, to allow placement of the cautery return electrode.
14. Remove all loose hair clippings and transfer the dam from the preparation area to the procedure cradle (Figure 2A). Position the animal in the supine position over the electrosurgical grounding electrode (Figure 2B). Because the electrode surface is similar in size to the animal, cover it with gauze, leaving only exposed the placement for the shaved dorsal contact area to ensure effective grounding while minimizing unnecessary skin contact.

***Note:*** Apply non-sterile ultrasound gel or lubricant between the trimmed dorsal area and the dispersive electrode to improve contact and ensure safe return of electrosurgical energy delivered through the guide.

15. Angle the cradle at 25-35° (geared head scale, where the vertical position is 90°) to elevate the head and reduce pressure on the inferior vena cava caused by the weight of the gravid uterus.
16. Create a sterile field by performing three alternating scrubs of the abdominal skin using 7.5% povidone-iodine solution (e.g., Betadine Surgical Scrub) and 70% ethanol. At each scrub, use a circular motion moving from the center outward.

**CRITICAL**: When creating the sterile field, use only the minimum volume of antiseptic solution necessary to avoid soaking the fur. Excess moisture can contribute to heat loss and hypothermia. Before initiating ultrasound-guided injections, confirm that physiological parameters have stabilized. After establishing the sterile field, ensure that only sterile or disinfected instruments come into contact with the prepared skin. These include the sterile guide, disinfected ultrasound transducer, and sterile tongue depressors or cotton-tipped applicators used to apply sterile gel or remove air bubbles as needed.

### Ultrasound-guided fetal injections

This section outlines the ultrasound-guided fetal injection procedure. The protocol combines stereotaxic mechanical control with real-time ultrasound imaging to enable selective targeting of the fetal brain. It describes the steps involved in (1) injection planning, (2) fetal targeting, and (3) controlled tissue penetration, along with troubleshooting guidance.

#### Timing: 5-20 min (per fetus)

17. Disinfect the ultrasound transducer by draping the transducer in sodium hypochlorite-based disinfectant wipes (e.g. Sani-Cloth Bleach, orange top) and allow it to remain in contact for 4 min to ensure sterilization.

***Note:*** Follow manufacturer’s guidelines to ensure compatibility of the disinfectant with the transducer.

18. Secure the ultrasound transducer in its custom-designed holder (Figure 2A).
19. Apply sterile ultrasound gel to the abdomen. Use a sterile tongue depressor to spread the gel evenly as needed, ensuring a layer thickness of approximately 1-2 cm to allow proper immersion of the angled transducer (Figure 2C).

***Note:*** To prevent or minimize heat losses in body temperature, warm the gel to 37°C (e.g., in incubator or warming pad) prior to application.

20. Position the angled ultrasound transducer against the abdominal skin within the sterile ultrasound gel layer (Figure 2C).

***Note:*** the US transduced holder is carried by a rotator mounted on a stereotaxic arm. Angle the transducer at an angle (30-50°) using either the arm or the rotation adapter (arm or adapter scales, where the vertical position is 0°) to allow space for the guide needle.

21. Mount the guide needle in the guide holder using sterile hemostatic forceps. Use the ball end hex driver 5/64" to loosen the screw holding the two guide holder plates and insert the guide needle between them.

***Note:*** The angle of the arm carrying the guide holder depends on the planned trajectory but is typically set between 35-45° to allow for the guide needle beneath the transducer (Figure 2C). The rotation adapter should be set at 0°.

22. Use the ultrasound transducer and the mechanical translation plates supporting the cradle to position the target fetus for injection. Use the geared head to adjust the dam’s position as needed to optimize fetal alignment for targeting.
23. Advance the guide needle through the ultrasound gel and align it to the ultrasound transducer over the fetal head (Figure 2C).
24. Adjust the angle of the guide tube by modifying the stereotaxic arm to align with the planned injection trajectory and optimize fetal targeting.

***Note:*** When determining the optimal injection trajectory, avoid nipples (in rats), maternal internal organs (e.g., bladder and intestines), and large vessels (e.g., umbilical cord). Whenever possible, avoid penetrating the placenta by repositioning the pregnant dam using the geared head and translation plates. If placental penetration is unavoidable for proper targeting, use color Doppler mode on the ultrasound system to identify major vessels and minimize the risk of hemorrhage along the needle trajectory. For most injection penetrations done through the placenta, no blood is observed upon withdrawal of the needle and guide. In rare cases, a minimal amount of blood may be observed during needle retraction, without evidence of active bleeding.

25. While gently pressing the guide needle against the skin, apply a brief electric current to the non-insulated blunt side of the guide to help tissue penetration with minimal displacement of the fetus (**Methods Video 4**).

***Note:*** Because the 12° bevel angle of the injection needle allows penetrating the abdominal wall and uterus, advancing the guide tube beyond the subcutaneous tissue layer is not necessary. We have tested injection needles with 12°, 30° and 60° bevel angles and observed smoother tissue penetration with the steeper angle.

**Troubleshooting 1**: The cautery step may introduce air bubbles at the site of skin penetration (**Methods Video 4**). In most cases, these bubbles cause minimal disruption of ultrasound visualization due to the angled approach, which displaces the bubbles outside the imaging field. If necessary, use a sterile cotton swab applicator to displace the bubbles from the field of view. To facilitate this step, keep the guide tube in place and retract the transducer using the z-axis of the stereotaxic arm. Once the gel is free of bubbles, reposition the transducer at the original imaging location.

26. Load virus to Hamilton syringe.

***Note:*** High levels of viral transduction are achieved using high-titer preparations (e.g., Addgene rAAV viral preparations ≥ 1x10^12^ vg/mL). The injected volume varies depending on gestational age at the time of injection and the construct (e.g., for rAAV maximum of 60 μL per fetus in marmosets and 10μL in rats lead to broad and dense transduction). Injection parameters should be empirically optimized for each experimental condition (e.g., viral vector, gestation stage) and species.

27. Smoothly advance the injection needle manually through the guide, penetrating the abdominal wall and uterine muscle, and continue into the uterine lumen adjacent to the fetal skull under ultrasound guidance.

***Note:*** With experience, manual advancement of the injection needle provides tactile feedback when traversing the different abdominal and uterine tissue layers. This sensory feedback can also assist in assessing the relative firmness of the developing fetal skull prior to injection.

**Troubleshooting 2**: The position and angle of the needle relative to the fetal head are critical. The optimal approach angle depends on skull thickness and the size of the uterine lumen, which vary by species and gestational age. Confirm needle entry into the abdominal wall or uterus by visualizing the increased echogenicity of the needle tip under ultrasound. If angle correction is required after tissue entry, use the rotator supporting the guide tube holder to perform fine angular adjustments. Alternatively, modify the angle of the cradle supporting the pregnant dam using the geared head or, for minor trajectory corrections that do not require a change in angle, reposition the target fetus by moving the dam with the translation stages.

28. Under ultrasound guidance, manually penetrate the fetal skull using a brief controlled jolt that advances the needle tip by approximately 1-3 mm (**Methods Video 5**).

**Troubleshooting 3**: Fetal skull penetration is most successful when attempted from an angle having maximum mechanical advantage, minimizing the risk of slipping along the convex skull surface (**Methods Video 6**), and in regions with thinner developing bone. In cases of trajectory misalignment or unsuccessful penetration, it is usually possible to readjust the geometrical approach to achieve successful entry without removal of the needle from the uterine lumen. This can be achieved by repositioning the animal using cradle adjustments while keeping the guide tube in place. Alternatively, modify the injection needle angle using the rotator supporting the guide tube holder. If realignment is not achievable with the needle within the uterine lumen, retract the needle to the subcutaneous space and readjust the trajectory. Complete removal of both the needle and guide is rarely required.

29. Manually inject the viral vector into the target region under ultrasound guidance.

***Note:*** To reduce backflow of rAAV following injection, maintain the needle in position for up to 5 min before slow withdrawal. Confirm successful targeting by visualizing the needle tip position under ultrasound. Introducing a small air bubble at the end of the injection can increase the echogenicity of the injectate, thereby facilitating visualization and confirmation of the target site.

***Note:*** Performing the procedure with two operators is helpful. While one operator identifies and positions the fetus and inserts the guide tube, the second operator can monitor vials and prepare and load the syringe. This approach reduces the time required per injection and shortens the overall duration of the procedure.

**Troubleshooting 4**: Following an injection, we occasionally observe uneven distribution of the viral vector within the CSF, resulting in preferential transduction of one hemisphere, regional differences in expression, or leakage outside the brain. Preferential delivery along the midline and slower vector administration and needle withdrawal, may improve vector distribution and reduce variability in transduction.

30. Withdraw the injection needle from the fetus and dam under ultrasound guidance.
31. Remove the guide tube from the dam skin.
32. Assess fetal viability by monitoring fetal heat rate under ultrasound to confirm stable heart function.

**Troubleshooting 5:** Transient changes in fetal heart rate were occasionally observed in rat fetuses at the earliest gestational stage tested, namely at PC13. In these cases, spontaneous recovery occurred without intervention. In subsequent procedures, the injected volume was reduced from 10 μL to 5 μL, after which similar changes were no longer observed.

33. Repeat steps 22 to 32 with the other fetuses.

***Note:*** Unless bent, the same guide needle can be reused across injections in different fetuses. It may also be reused across procedures following thorough cleaning with acetone and subsequent disinfection with 70% ethanol.

### Post-procedure monitoring and recovery

This section outlines the post-procedural care steps following completion of the ultrasound-guided injection procedure.

#### Timing: 15-25 min

34. Remove the ultrasound gel from the abdominal surface using sterile gauze.
35. Apply a small amount of local anesthetic (e.g., lidocaine) to each skin entry site to reduce discomfort, followed by a triple antibiotic ointment to minimize the risk of infection.
36. Allow the pregnant dam to recover in a warm environment prior to returning it to the housing room.
37. Once the animal is bright, alert, and responsive, administer a small volume of dextrose (marmoset: 0.1-0.2 mL, s.l.; rat: 0.1 mL, s.l.) as needed to prevent or treat hypoglycemia and support post-procedural recovery. Transfer the dam to the housing room only after full recovery is confirmed.

***Note:*** In marmosets, monitor animals at least once daily for 7 days following the intrauterine injection procedure for signs of abdominal pain, uterine contractions, or vaginal discharge.

### Postnatal identification of transduced animals

This section outlines the procedures used for in vivo identification of transduced animals from birth.

#### Timing: 10-20 min

38. In a dark environment, use a fluorescence flashlight equipped with high-intensity LEDs and the appropriate excitation filter set to detect reporter expression through the skull and/or skin. For green-shifted reporters, an option is the model Xite-RB (Nightsea) with a Royal Blue excitation filter (440–460 nm) and 500–560 nm bandpass barrier filter glasses. For red-shifted reporters, model Xite-GR (Nightsea) with a Green excitation filter (510–540 nm) and 600 nm bandpass barrier filter glasses.

***Note:*** Transgene expression persists at high levels into adulthood and remains detectable in hairless regions. However, identification of transduced animals is most reliable during the perinatal period.

**Troubleshooting 6**: In rats, reporter expression is often most prominent in the brain, spinal cord, back musculature, and facial regions due to peripheral nerve labeling. In marmosets, expression is typically most readily observed in less pigmented and hairless regions. Reporter expression may not always be readily detectable at birth, depending on the viral construct, promoter strength, titer, injection volume, and anatomical distribution of transduction. In such cases, careful selection of viral constructs and the use of auxiliary markers, such as intramuscular AAV injections, co-injected reporter vectors, or vital dyes, may facilitate postnatal identification of transduced animals.

## Expected outcomes

FIVI can be successfully performed across a broad gestational window. In marmosets we have performed FIVI from PC62 and in rats from PC121. Successful procedures are characterized by stable maternal and fetal physiological parameters throughout the intervention, maintenance of fetal cardiac activity following injection, and rapid recovery of the dam. In our experience, pregnancies proceed to term within the expected gestational timeframe, and offspring are delivered without complications, such as dystocia. Only rarely has FIVI resulted in miscarriage. Further discussion of procedural outcomes is provided in Ribeiro Gomes et al. (2026).

**Figure 5.**
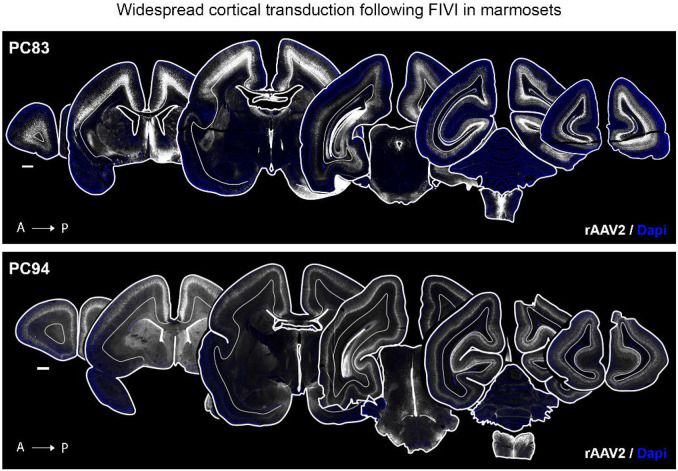
Fetal intracerebral delivery of rAAV leads to extensive transduction of cortical neurons in marmosets. Representative marmoset coronal sections illustrating extensive cortical transduction across anterior-to-posterior regions following FIVI performed at two developmental stages and analyzed at postnatal day 0. Broad cortical transduction is achieved following injections at both gestational ages, demonstrating the feasibility of FIVI-mediated gene delivery across developmental windows. The examples shown are from rAAV2 FIVI. FIVI volume: 40 μL at PC83 and PC94. Scale bars: 1 mm.

Figure 5 shows successful transduction following FIVI performed at two developmental stages, PC83 and PC94. The representative examples shown here were generated using rAAV2, but similar widespread transduction patterns have also been observed using AAV9 and AAV8 (Ribeiro Gomes et al., 2026). This highlights a key feature of the approach: broad transduction is a general result of fetal CSF delivery rather than a property unique to a specific rAAV serotype.

**Figure 6.**
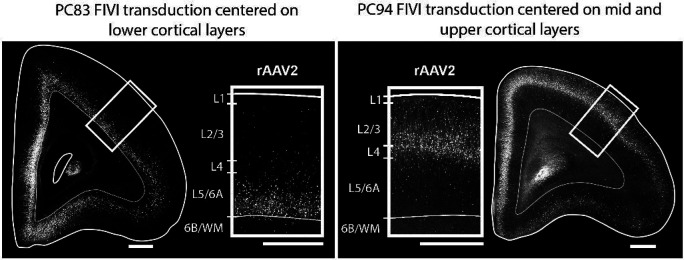
Gestational age influences transduction patterns following FIVI-mediated delivery of rAAV. Higher-magnification views of the prefrontal cortex from the animals shown in Figure 5. Transduced cells following PC83 FIVI are predominantly located in layers 5 and 6, whereas cells transduced following PC94 FIVI are predominantly located in layers 2 to 4. These differences illustrate how the prolonged gestation of marmosets enables temporal control of transgene delivery and cell-type targeting.

Another key feature of rAAV delivery into fetal marmosets is the increased temporal resolution for cell targeting afforded by their prolonged gestation (143 days). Figure 6 illustrates how injection timing influences transduction patterns, with earlier injections preferentially labeling neurons in deeper cortical layers and later injections labeling progressively more superficial populations. Cell populations permissive to rAAV transduction at earlier developmental stages are no longer efficiently transduced at later time points. Although the mechanisms underlying this developmental tropism remain unclear, our working hypothesis is that transduction efficiency depends on the maturation state of the targeted cells. Similar gestation-dependent patterns are observed in rats, although they are less pronounced because of the substantially shorter gestational period (23 days).

Further discussion of these developmental transduction patterns and additional strategies for targeting cell populations are provided in Ribeiro Gomes et al. (2026).

## Limitations

### Limitation 1:

The number of embryos that can be successfully injected is constrained by intrauterine space and fetal positioning. In rat dams, the number of fetuses injected typically ranges from 5 to 9 per litter, depending on gestational stage and uterine accessibility. At earlier gestational ages, the uterine horns are frequently positioned beneath maternal internal organs, limiting visualization and access. At later gestational stages, reduced intra-abdominal space may result in superimposed fetuses or positioning beneath maternal organs, further restricting access. Additionally, ultrasound image quality decreases for deeper fetuses, which may prevent reliable targeting. Although rare, similar limitations are observed in marmosets during advanced pregnancies, where deeper fetal positioning can prevent injection of all fetuses within a litter. In both species, fetal orientation may also limit targeting. Although repositioning the pregnant dam using the mechanical degrees of freedom of the cradle often improves access, certain anatomical constraints, such as the presence of the umbilical cord within the planned injection trajectory, may require exclusion of specific fetuses to avoid injury.

### Limitation 2:

The number of fetuses injected per procedure is also constrained by the procedure length. In both rat and marmoset dams, the total number of fetuses that can be injected is limited by the duration of anesthesia. To minimize potential adverse effects associated with prolonged anesthesia, including impacts on fetal development and maternal recovery, the total procedure time is restricted to 2 hours or less.

### Limitation 3:

Variability in viral transduction. Although efficient and widespread rAAV transduction can be achieved following FIVI, variability in distribution may occur. For instance, control of the vector backflow and dispersion outside of fetal skull is limited. In some cases, transduction may be weaker or more prominent unilaterally or relatively higher in the spinal cord than in the brain, depending on viral diffusion and injection dynamics.

## Troubleshooting

### Problem 1:

Poor ultrasound visualization of the fetus (refer to steps 12, 19, 25, 27 and 28).

### Potential solution:

- Ensure adequate removal of the dam’s hair (e.g., trimming and/or depilation) to improve ultrasound image quality.
- Ensure sufficient sterile gel thickness (approximately 1–2 cm for angled transducer immersion) to cover the base of the transducer.
- Displace air bubbles introduced during application or cautery using a sterile cotton-tipped applicator or tong depressor.
- Reposition the dam using the translation plates and geared head to improve fetal alignment.
- Reapply gel if necessary to restore acoustic coupling.

### Problem 2:

Placenta or umbilical cord obstructing injection trajectory (refer to steps 24, 27 and 28)

### Potential solution:

- Use color Doppler mode to identify and avoid major vessels along the planned path.
- Reposition the dam using the geared head to displace the placenta or umbilical cord out of the field of view.
- Modify trajectory to introduce the needle in the amniotic sac lumen near the injection target. Then use the translation stages or stereotaxic arm movement to slowly approach the needle to the target while displacing the vessel away from the field of view and planned trajectory. Do so without contacting the vessel with the injection needle bevel.
- When safe trajectory cannot be achieved without compromising a blood vessel, omit injection of that fetus to prevent injury. If transplacental injection is required, proceed with caution and closely monitor for bleeding under ultrasound visualization.

### Problem 3:

Difficulty penetrating the fetal skull (refer to steps 28).

### Potential solution:

- Perform skull penetration at an angle that maximizes mechanical advantage to reduce slipping along the convex skull surface, and preventing misplacement of the fetus outside of the ultrasound image. Adjust the injection angle using the rotator supporting the guide tube holder.
- Reposition the dam using the translation stages and geared head to optimize alignment and maintain the fetus within the ultrasound field of view.
- When possible, plan the injection trajectory through regions of thinner developing bone to facilitate penetration.

### Problem 4:

Reduced fetal survival following injection (refer to step 24, 29, 32 and 33).

### Potential solution:

- Optimize injected volume and viral titer according to gestational stage and viral construct. Excessive volume or high titer may increase physiological stress and reduce survival.
- Minimize total procedure time and ensure stable physiological parameters throughout the procedure (e.g., adjust isoflurane concentration to the lowest effective level for each animal to maintain stable anesthesia).
- Confirm proper trajectory planning to avoid major vessels and excessive tissue disruption.

### Problem 5:

Transient changes in fetal heart rate following injection (refer to step 32).

### Potential solution:

- Monitor fetal cardiac activity under ultrasound immediately after injection to assess stability.
- Reduce the injected volume if bradycardia or irregular heart rate is observed.
- Allow time for spontaneous recovery before proceeding with additional injections.

### Problem 6:

Difficulty identifying injected neonates (refer to step 38).

### Potential solution:

- Reporter expression may not be readily detectable at birth depending on the viral construct (e.g., promoter strength), titer, injected volume, and anatomical distribution. In such cases, consider co-injecting a complementary vector or serotype expressing a readily detectable reporter to facilitate postnatal identification in rats and marmosets. Co-injection with a vital dye, such as Fluorescein, Indigo Carmine, Evans blue, can also be used in rats.
- Intramuscular AAV injection at the time of the fetal procedure can serve as an auxiliary marker to assist in retrieving injected neonates in both species.
- CSF injection of viral vectors may result in transduction of the peripheral nervous system. In such cases, peripheral optogenetic stimulation (e.g., of the tail) in animals expressing opsins may evoke a motor response and/or vocalization, which can aid in identifying transduced animals.

### Problem 7:

Uneven or weak viral transduction of the fetus (refer to step 29).

### Potential solution:

- Prioritize midline injections when feasible to promote more uniform viral diffusion.
- To minimize backflow, maintain the needle in position for up to 5 min before withdrawal and retract the needle slowly and steadily.
- Confirm that injection volume and viral titer are appropriate for the gestational stage. Insufficient volume or low titer may result in reduced or sparse transduction.
- Variability can be managed by screening animals postnatally. When expression levels are sufficiently robust at birth, uneven transduction patterns can be qualitatively assessed using a handheld light source, and animals may be assigned to experiments accordingly.

## Resource availability

### Lead contact

Further information and requests for resources and reagents should be directed to and will be fulfilled by the lead contact, David A. Leopold (leopoldd@mail.nih.gov).

### Technical contact

Further information on executing this protocol should be directed to and will be answered by the technical contact, Ana Rita Ribeiro Gomes (anarita.ribeirogomes@nih.gov).

### Materials availability

Materials used in this protocol are listed in the Key Resources table.

### Data

All data reported in this paper will be shared by the lead contact upon request.

### Code

This paper does not report original code.

### Additional information

Any additional information is available from the lead contact upon request.

## Supplementary Material

Data S1. Drawings of the guide tube holder and cradle.

## Figures and Tables

**Methods Video 1:** Guide needle sheathing by shrinking Palladium Pebax tubing with a heat gun. The video shows angled positioning of the guide needle with hemostatic forceps and progressive heat application from the beveled to the blunt end of the needle. Tubing contraction and proximal retraction are visible throughout the process and become more apparent during final contraction at the blunt end.

**Methods Video 2.** Ultrasound imaging of a marmoset fetal head acquired using the Vevo MD ultrasound system with the UHF22 transducer. Scale: 3 mm.

**Methods Video 3.** Ultrasound imaging of the same fetus shown in Methods Video 2, acquired using the Clarius L20 HD3 handheld ultrasound system. Scale: 3 mm.

**Methods Video 4.** Skin penetration facilitated by current pulses applied to the blunt end of the guide tube. Note the transient reduction in echogenicity beneath the guide tube caused by air bubble formation during electrocautery. Ultrasound visualization can be improved by displacing or removing the bubbles. Scale: 1 mm.

**Methods Video 5.** Ultrasound-guided FIVI targeting the midline third ventricle in a PC107 marmoset fetus. The injection trajectory traverses the placenta before entry into the fetal brain through a weaker region of the developing skull. Scale: 1 mm.

**Methods Video 6.** Displacement of the fetal head during attempted skull penetration at PC80. During retraction of the needle, the fetus was repositioned by moving the dam with the translation stages, thereby shifting the fetus toward the left. Scale: 1 mm.

**Table:** Key resources table

REAGENT or RESOURCE	SOURCE	IDENTIFIER
**Bacterial and virus strains**		
AAV2-CAG-GFP	Addgene	37825-AAV2
**Chemicals, peptides, and recombinant proteins**		
Atropine Sulfate Injection, 0.54 mg/mL	MWI	NDC 13985-354-04
Meloxicam Injection USP, 5 mg/mL	Meloxivet	NDC 17033-051-10
Diazepam Injection, 5 mg/mL	Multiple sources	N/A
Isoflurane	Fluriso	NDC 13985-528-40
Hair removal creme (Nair)	Church & Dwight Co., Inc.	N/A
Ophthalmic Ointment (0.4% Sodium Hyaluronate)	Ophthavet	NDC 17033-047-05
Ethanol wipes	Multiple sources	N/A
Povidone iodine	Purdue Products LP	NDC 67618-154-16
Lidocaine Ointment USP, 5%	Multiple sources	N/A
Water-based lubricant	Multiple sources	N/A
Non-sterile ultrasound gel	Multiple sources	N/A
Aquasonic 100 - Sterile Single Use - Overwrapped Foil Pouches 48 per box	Aquasonic	PLI 01-01
**Experimental models: Organisms/strains**		
Sprague-Dawley rats	Charles River	Strain Code: 001
Common marmosets (Callithrix jacchus)	NIMH	N/A
**Software and algorithms**		
Fiji	ImageJ	https://imagej.net/software/fiji/
Adobe Photoshop 2026	Adobe	https://www.adobe.com/products/photoshop.html
**Other**		
RC2 Rodent Circuit Controller	VetEquip	922100
Induction Chamber, 7 liter	VetEquip	941488
Color coded, nylon-reinforced, 1/4" ID, conductive gas supply hose	VetEquip	931503
12mm Nosecone and 14mm Nosecone	VetEquip	921612 and 921614
Anesthesia silicone mask, reusable, size 00, premature baby	Drager	MP02900
NORMOTHERM^™^ INFRARED HEATER	Britz & Company	N/A
SomnoSuite^®^ Low-Flow Anesthesia System	Kent Scientific Corporation	SS-01
PhysioSuite^®^	Kent Scientific Corporation	PS-02
IntelliVue MX500 Patient Monitor	Philips Medizin Systeme Böblingen GmbH	866064
Micro NeoLead, AAMI radio lead	Philips Medical Systems Hsg	989803183141
Nasal Alar SpO2 Sensor	Philips Medical Systems Hsg	989803205381
NIBP Cuffs (Neonatal single-patient cuff size #1)	Philips Medical Systems Hsg	M1866B
Symmetry Pro-120 Multi-Pupose Electrosurgical Generator	Bovie	A1250S
Push Button Electrosurgical Pencil 50/Box	Bovie	ESP1
Reusable Grounding Cable	Bovie	A1252C
Disposable Split Adult Return Electrodes	Bovie	ESRE-1
23 gauge, Hubless Needle, custom length (2 in), point style 4, 12 DEG, 6/PK	Hamilton Company	22023-01
24 gauge, Hubless Needle, custom length (2 in), point style 4, 12 DEG, 6/PK	Hamilton Company	22024-01
33 gauge, Small Hub RN Needle, custom length (2.5 in), point style 4, 12 DEG, 6/PK	Hamilton Company	7803-05
5 μL, Model 75 RN Syringe, Needle Sold Separately	Hamilton Company	7634-01
10 μL, Model 701 RN Syringe, Needle Sold Separately	Hamilton Company	7635-01
50 μL, Model 710 RN Syringe, Needle Sold Separately	Hamilton Company	7637-01
100 μL, Model 710 RN Syringe, Needle Sold Separately	Hamilton Company	7638-017634-01
Curved hemostatic forceps	Multiple sources	N/A
Ball end hex driver 5/64"	Multiple sources	N/A
Ball End Hex Driver 3/32"	Multiple sources	N/A
Pipette tips 20 μL, filtered	Multiple sources	N/A
Pipette tips 100 μL, filtered	Multiple sources	N/A
Pipette, single-channel, volume 20 μL	Multiple sources	N/A
Pipette, single-channel, volume 100 μL	Multiple sources	N/A
Palladium^™^ "Pebax^™^" Heat Shrink Tubing	Component Supply	PBST2-040-40-004C7635-01
1500 Watt 10 Amp 12 Temperature Heat Gun	Harborfreight	69343
18.70 mm A/P Bar	KOPF	N/A
A.P. Slide Attachment	KOPF	Model 1261 A/P Slide Attachment
Rotation Adapter	KOPF	Model 1460-G Rotation Adapter
Travel Vertical Translation Stage	KOPF	VAP4/M
Stereotaxic arm and micro manipulator with fine adjustment	KOPF	Model 1460-61
Single Axis Translation Stage	THORLABS	LT1/M
Cube Geared Head	Arca-Swiss	8501303.1
Aluminum Breadboard	THORLABS	MB2530/M
Animal cradle	NIMH/SI	N/A
Stereotaxic arm base holder	NIMH/SI	N/A
Ultrasound probe holder	NIMH/SI	N/A
Vevo MD Imaging System	Fujifilm Sonosite, Inc.	51475
UHF22 Transducer	Fujifilm Sonosite, Inc.	51414
UHF48 Transducer	Fujifilm Sonosite, Inc.	51415
Hair trimmer	WAHL	41590-0438
Hair trimmer blade	WAHL	41590-7840
Heat Pad - Large	Arbor Scientific	P3-1015-03
2x2 Gauze	Multiple sources	N/A
4x4 Gauze	Multiple sources	N/A
Tongue depressors	Multiple sources	N/A
Sterile tongue depressors	Multiple sources	N/A
Cotton Swab	Multiple sources	N/A
Sterile cotton swab	Multiple sources	N/A
Tape	3M	1527-1
